# NRF2 and Key Transcriptional Targets in Melanoma Redox Manipulation

**DOI:** 10.3390/cancers14061531

**Published:** 2022-03-16

**Authors:** Evan L. Carpenter, Alyssa L. Becker, Arup K. Indra

**Affiliations:** 1 Department of Pharmaceutical Sciences, College of Pharmacy, Oregon State University, Corvallis, OR 97331, USA; carpenev@oregonstate.edu (E.L.C.); beckera3@hawaii.edu (A.L.B.); 2 John A. Burns School of Medicine, University of Hawaii, Honolulu, HI 96813, USA; 3 Knight Cancer Institute, Oregon Health & Science University, Portland, OR 97239, USA; 4 Department of Biochemistry and Biophysics, Oregon State University, Corvallis, OR 97331, USA; 5 Linus Pauling Science Center, Oregon State University, Corvallis, OR 97331, USA; 6 Department of Dermatology, Oregon Health & Science University, Portland, OR 97239, USA

**Keywords:** NRF2, glutathione, thioredoxin, peroxiredoxin, NQO1, HO-1, HSP70, SOD, melanoma, antioxidant

## Abstract

**Simple Summary:**

Melanocytes are the pigment-producing cells in the skin that help to protect it against damaging solar ultraviolet radiation. As they engage in this function, they are subject to an inordinate amount of oxidative stress. To protect themselves, they utilize numerous antioxidant systems to reduce the amount of reactive oxygen and nitrogen species present in the cells, and this activity then contributes towards the prevention of cancer formation. However, after the formation of melanomas these same antioxidant systems are often co-opted by the cancer in order to promote its uncontrolled growth and metastasis. The purpose of this review is to highlight how the melanocyte’s antioxidant systems, regulated by the transcription factor NRF2 and its targets, are co-opted in melanomas and, therefore, could be targeted for novel therapies to treat melanomas going forward.

**Abstract:**

Melanocytes are dendritic, pigment-producing cells located in the skin and are responsible for its protection against the deleterious effects of solar ultraviolet radiation (UVR), which include DNA damage and elevated reactive oxygen species (ROS). They do so by synthesizing photoprotective melanin pigments and distributing them to adjacent skin cells (e.g., keratinocytes). However, melanocytes encounter a large burden of oxidative stress during this process, due to both exogenous and endogenous sources. Therefore, melanocytes employ numerous antioxidant defenses to protect themselves; these are largely regulated by the master stress response transcription factor, nuclear factor erythroid 2-related factor 2 (NRF2). Key effector transcriptional targets of NRF2 include the components of the glutathione and thioredoxin antioxidant systems. Despite these defenses, melanocyte DNA often is subject to mutations that result in the dysregulation of the proliferative mitogen-activated protein kinase (MAPK) pathway and the cell cycle. Following tumor initiation, endogenous antioxidant systems are co-opted, a consequence of elevated oxidative stress caused by metabolic reprogramming, to establish an altered redox homeostasis. This altered redox homeostasis contributes to tumor progression and metastasis, while also complicating the application of exogenous antioxidant treatments. Further understanding of melanocyte redox homeostasis, in the presence or absence of disease, would contribute to the development of novel therapies to aid in the prevention and treatment of melanomas and other skin diseases

## 1. Introduction

Melanoma is the deadliest form of skin cancer and the leading cause of death due to skin disease [1]. It has exhibited an increased incidence in the United States, with an average annual percentage change (AAPC) of 2.2% in males and 2.3% in females between 2013 and 2017. Fortunately, the rate of death between 2013 and 2018 has started to trend downward with an AAPC of −5.7% and −4.4% in males and females, respectively [2]. These decreases are likely due to a combination of more successful prevention and early detection strategies, as well as the development of increasingly efficacious therapies such as targeted BRAF or MEK inhibitors, immunotherapy, and all combinations thereof.

Melanomas arise from the malignant transformation of melanocytes, which are dendritic, pigment-producing cells located in the basal layer of the epidermis, hair follicles, inner ear, and uvea of the eye [3]. Normally, melanocytes are tasked with the protection of adjacent skin cells from solar ultraviolet radiation (UVR) by synthesizing and distributing photoprotective melanin pigment [4]. However, melanocytes are still susceptible to UV-induced DNA damage, which can lead to the formation of key driver mutations in oncogenes, including proliferation/survival regulators such as *BRAF* and *NRAS* and cell-cycle regulators such as *CDK4* [5,6,7]. Additionally, UVR also leads to the rapid accumulation of passenger mutations, which might not be drivers of melanomagenesis themselves, but collectively serve to cause heightened tumor heterogeneity that complicates treatment [8]. Upon metastasis, melanomas are highly aggressive, often migrating to the brain and lungs, and five-year survival rates are 19% for patients diagnosed with stage IV disease [9].

Current treatment modalities for melanoma include surgical resection, radiotherapy, chemotherapy, immunotherapy, biochemotherapy, and targeted therapy [10]. These agents may be used alone or in combination depending on the tumor’s stage, location, and genetic profile, as well as the patient’s goals for treatment. Earlier stage disease (stages I-IIIB) can usually be fully resected [11]. Surgical margins depend on the depth of invasion: 0.5 cm for in situ melanomas, 1 cm for tumors ≤2 mm in thickness, and 2 cm for tumors >2 mm in thickness [10,12]. Surgical excision (metastasectomy) is also part of the standard of care in the treatment of a solitary site of metastasis. Radiotherapy, although rarely used in treatment of the primary tumor, can also be used for metastatic lesions, especially of the skin, bone, and brain [13]. In high-risk melanomas (stages IIC-IV), adjuvant therapy is recommended following a complete resection of the primary tumor [14].

Untargeted chemotherapies such as dacarbazine and temozolomide were the first pharmaceutical options available for advanced melanomas; however, they have been largely unsuccessful and typically do not improve overall survival [15]. Resistance to apoptosis is thought to be the major mechanism of chemotherapeutic drug failure in melanomas [16]. Untargeted chemotherapies have largely been replaced by newer options but may still have some utility in palliative treatment [15].

Approximately 70% of patients with cutaneous melanoma have oncogenic mutations in signaling pathways involving cell proliferation; targeted therapeutic strategies use small molecule inhibitors or antibodies that affect these mutated proteins [17]. FDA-approved targeted therapies for melanoma include BRAF^V600E^ and MEK inhibitors. Compared to chemotherapy, BRAF inhibitors improve clinical response rates, progression-free survival, and overall survival in metastatic melanoma patients with *BRAF* mutations [18,19]. However, their efficacy can be limited due to the rapid development of multiple mechanisms of resistance [20]. In addition to possible drug resistance, BRAF inhibitor monotherapy can also be associated with cutaneous toxicities secondary to paradoxical MAPK pathway activation [19,21]. Combined therapeutic strategies are utilized to attempt to overcome these issues. Targeting signals downstream of BRAF, such as MEK, decreases MAPK-driven acquired resistance and toxicity, resulting in a greater duration of any response to therapy and fewer side effects [22,23]. While combined anti-BRAF/MEK therapy is initially highly effective in patients with melanomas harboring mutant *BRAF*, eventual resistance is not uncommon [24,25,26,27,28].

Improved understanding of the pathophysiology of the role of the immune system in tumorigenesis has led to the development and approval of several immunotherapies that show promising efficacy [29,30,31]. Combination immunotherapies show even greater promise; the CheckMate 067 trial demonstrated that, among patients with advanced melanoma, sustained long-term survival was seen in a greater percentage of patients who received nivolumab (anti-PD-1) plus ipilimumab (anti-CTLA-4) compared to ipilimumab alone [32,33]. Remarkably, nivolumab/ipilimumab combination therapy is the only currently available treatment for metastatic melanoma with a median overall survival greater than five years [33]. While immunotherapies show great promise, it is possible for primary and acquired resistance to occur, especially in the case of monotherapy, which may be due to the lack of recognition by T-cells, interactions with the cancer immune cycle, and/or complex signaling pathways within the tumor microenvironment [34,35,36].

Biochemotherapy combines the apoptotic and DNA-damaging mechanisms of chemotherapy with the immunomodulatory effects of immunotherapy [10]. Compared to chemotherapy monotherapy, biochemotherapy improves median progression-free survival but does not improve overall survival and can be associated with severe toxicity [10]. Therefore, further understanding of the metastatic progression of melanomas could provide insight into novel therapies that contribute to prevention and treatment.

Oxidative stress is an important aspect involved in normal melanocyte physiology, melanoma initiation, and disease progression, as well as a potential source for novel therapies going forward. Even in the absence of disease, melanocytes are subject to a significant degree of oxidative stress that originates both exogenously, from sources such as UVR, and endogenously, from metabolic pathways including mitochondrial respiration and melanogenesis [37,38,39]. These sources of oxidative stress can then contribute towards the regulation of nuclear factor erythroid 2-like 2 (*NFE2L2* encoding NRF2) related signaling pathways [40].

Notably, UV irradiation of melanocytes in vitro has been shown to induce the activation of NRF2 and the subsequent expression of antioxidant target genes including *GCLM*, *GCLC*, *NQO1*, and *HMOX1* [41]. Furthermore, in vivo human skin UV irradiation has been shown to induce the expression of another NRF2 target, thioredoxin reductase 1, in melanocytic nevi [42]. We have also shown, using a tissue-specific knockout mouse model, that melanocytic thioredoxin reductase 1 contributes to both melanocyte development and photobiology, as we observed pigmentation defects and elevated UV-mediated 8-oxo-2′-deoxyguanosine DNA damage in its absence [43]. Overall, NRF2 and its antioxidant target genes are important to normal melanocyte physiology and the response to UVR and act as tumor suppressors in this context.

As with many cancers, melanomas exhibit elevated oxidative stress both intracellularly as well as in the tumor microenvironment [44,45]. This elevation is due, in part, to altered metabolic pathways that cancer cells engage to sustain their dysregulated proliferation, which NRF2 contributes to [46,47,48]. Classically, increased oxidative stress has been held to simply contribute to melanoma initiation and progression by inducing a microenvironment conducive to tumor heterogeneity and metastasis. This is due to the increased mutational burden, as well as the activated signaling pathways associated with heightened tissue damage, such as inflammation [49]. It would then follow that exogenous antioxidant therapies should be highly efficacious in reducing proliferation and progression. However, it is currently understood that the role played by oxidative stress in melanomas is highly complex, as cancer cells establish a reprogrammed redox homeostasis that enables the tumor-promoting aspects of oxidative stress while avoiding its associated toxicity [50]. This is often seen by the activation of NRF2 in melanomas, particularly in those harboring *KEAP1* mutations, during progression with the upregulation of key downstream redox regulatory factors including elements of the glutathione (GSH) system, thioredoxin (TRX) system, cysteine cycling, nicotinamide adenine dinucleotide phosphate (NADPH): quinone oxidoreductase 1 (NQO1), heme oxygenase 1 (HO-1), heat shock protein 70 (HSP70), and peroxiredoxins [51,52,53,54,55,56,57,58,59,60,61].

Furthermore, the complexity of this reprogrammed redox homeostasis also explains the lack in efficacy often exhibited by exogenous antioxidant treatments thus far [62]. Altogether, it is certain that further research into how oxidative stress contributes towards melanocyte physiology and melanoma pathophysiology is needed for potential anti- or pro-oxidant therapies to come to fruition. This review primarily focuses on the NRF2 pathway and potential transcriptional targets controlling redox signaling in melanomas.

## 2. NRF2/KEAP1/ARE Signaling

### 2.1. NRF2 Protein Structure

**Figure 1:**
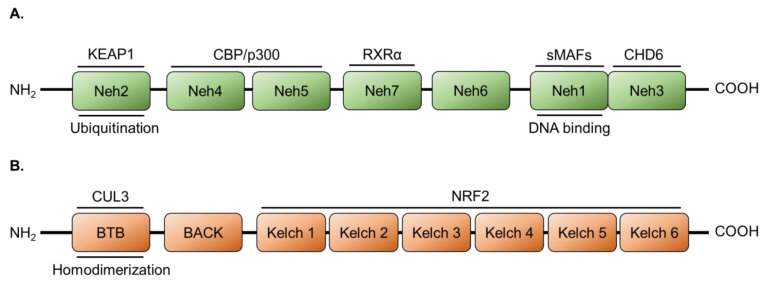
Scheme of the domains present in (**A**) NRF2 and (**B**) KEAP1 and associated functions. Above each protein domain are referenced interactions and below are associated functions. CBP, CREB binding protein; CHD6, chromodomain-helicase-DNA binding 6; CUL3, E3 ubiquitin ligase cullin-3; KEAP1, kelch-like ECH-associated protein 1; Neh, NRF2-ECH homology; NRF2, nuclear factor erythroid 2-like 2; RXRα, retinoid x receptor α; and sMAF, small musculoaponeurotic fibrosarcoma.

NRF2 is a cap’n’collar basic leucine zipper transcription factor comprised of seven NRF2-ECH homology (Neh) domains [63]. Each of these Neh domains has distinct functions that include DNA binding, transactivation activity, and binding sites for regulatory factors (Figure 1A). The foremost of the latter is the negative regulator, kelch-like ECH-associated protein 1 (KEAP1), which controls the subcellular localization and degradation of NRF2 through the Neh2 domain [64]. DNA binding is mediated by the Neh1 domain, which is also involved in NRF2 heterodimerization with transcription co-factors including small musculoaponeurotic fibrosarcoma (sMAF) [65,66]. Neh3 contributes towards NRF2 transactivation activity via the binding of chromodomain-helicase-DNA binding 6 (CHD6) to engage in chromatin remodeling necessary for transcription, and the other Neh domains bind assorted co-activators/co-repressors [67,68]. The nuclear receptor, retinoid X receptor α (RXRα), acts as a co-repressor binding to Neh7. Neh4/5 bind the histone acyltransferase and co-activator, cyclic-AMP response element-binding protein (CREB) binding protein (CBP, also CREBBP) [69,70]. Recently, another histone acyltransferase homologous to CBP, p300, has also been reported to bind the Neh4/5 domains and function similarly as a co-activator [71].

### 2.2. Regulation of NRF2 by KEAP1/CUL3

**Figure 2:**
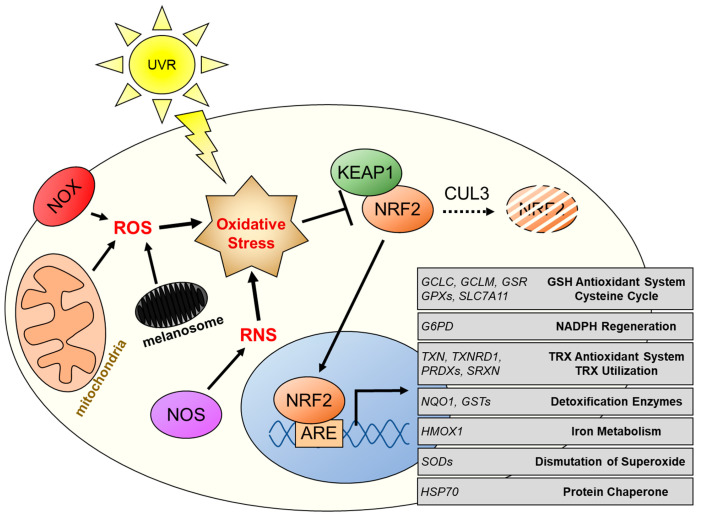
Sources of oxidative stress in melanocytes and subsequent activation of the NRF2/KEAP1/ARE-signaling pathway. Endogenous sources of oxidative stress include ROS produced by mitochondrial respiration, melanosomal melanogenesis, and the activity of NOX enzymes as well as RNS produced by NOS enzymes. It should be noted that NOX enzymes are expressed in several subcellular compartments in addition to the plasma membrane, as depicted here. Exogenous sources of oxidative stress include environmental stressors such as UVR and exposure of the skin to pro-oxidant chemical substances. Under normal conditions, there is a consistent low level of NRF2 present that is sequestered in the cytosol by KEAP1. CUL3 can then bind to the NRF2/KEAP1 complex leading to the ubiquitination and subsequent degradation of NRF2. However, under conditions of oxidative stress, there are redox-sensitive cysteine residues on KEAP1 that become modified leading to the release of NRF2. Released NRF2 can then enter the nucleus, bind to AREs, and promote the expression of antioxidant genes. ARE, antioxidant response element; CUL3, E3 ubiquitin ligase cullin-3; GSH, glutathione; KEAP1, kelch-like ECH-associated protein 1; NOS, nitric oxide synthase; NOX, NADPH oxidase; NRF2, nuclear factor erythroid 2-like 2; RNS, reactive nitrogen species; ROS, reactive oxygen species; TRX, thioredoxin; and UVR, ultraviolet radiation.

The KEAP1 protein consists of three functional domains: a broad-complex, tramtrack and bric a brac (BTB) domain; the BTB and c-terminal kelch (BACK) domain; and six kelch motifs (Figure 1B). These kelch motifs form a β-propeller structure and are responsible for NRF2 binding [72]. It is at the BTB domain that KEAP1 forms a homodimer, as well as acting as an adaptor for another key NRF2 regulator, E3 ubiquitin ligase cullin-3 (CUL3) [73]. Under normal physiological conditions, there are low levels of NRF2 localized to the cytosol, where it is bound and sequestered by KEAP1. Recruitment of CUL3 to the complex leads to the KEAP-1-mediated poly-ubiquitination of lysine residues within the Neh2 domain of NRF2, which is then followed by degradation through the ubiquitin proteasome system (Figure 2).

In the event of elevated oxidative stress, sensor cysteine residues located in the KEAP1 kelch domains act as a redox switch, which is flipped upon modification by oxidants. In this event, NRF2 is disassociated from the KEAP-CUL3 complex and accumulates in the cytosol [73]. It is then able to localize into the nucleus where it heterodimerizes with sMAF transcriptional co-factors and binds to genomic antioxidant response elements (AREs) [65]. NRF2 binding of ARE sequences located in the promoter regions of target genes leads to induced expression [74,75].

### 2.3. Activation of NRF2 in Melanomas

In melanomas, tumor transformation is often driven by the dysregulation of the mitogen-activated-protein-kinase (MAPK)-proliferation signaling pathway as a consequence of *BRAF* and *NRAS* mutations seen in 50% and 20% of cases, respectively [7,8]. Recently, a direct connection has been made between the activation of NRF2 and MAPK signaling in melanomas. Jessen and collaborators reported that the knockdown of NRF2 in melanoma cell lines led to decreased proliferation and *EGFR* expression [76]. Furthermore, the injection of NRF2-knockout mouse melanoma cells subcutaneously into C57BL/6 mice resulted in 40% of mice with no tumor formation as opposed to all mice injected with control cells. Mice with NRF2-knockout melanomas also exhibited a delayed tumor onset and prolonged tumor-free survival. They also demonstrated that NRF2 negatively regulated microphthalmia-associated transcription factor (MITF) activity, with NRF2 knockdown leading to induced melanogenesis gene expression associated with melanocyte differentiation. In a follow-up study, the same group reported that NRF2 regulates *EGFR* expression via binding to an ARE in its promoter region in melanoma cells [77]. MAPK signaling has also been shown to induce NRF2 activation in primary mouse cell lines with lentiviral transduction of oncogenic KRAS and BRAF [78]. The connection between NRF2 and EGFR signaling has been demonstrated in other cancer types as well [79,80].

The presence of elevated oxidative stress in melanomas is another cause of NRF2 activation during melanoma initiation and progression. As previously mentioned, the sources for this oxidative stress include UVR, melanogenesis, and reprogrammed cancer metabolism (Figure 2). Other important sources of ROS (e.g., superoxide and hydrogen peroxide) and RNS (e.g., nitric oxide) are associated with NADPH oxidase (NOX) and nitric oxide synthase (NOS) enzymes, respectively [50,81]. In melanomas, NOX1, NOX4, and NOX5 have been confirmed to be expressed, with NOX1 expression consistent throughout progression and NOX4 upregulated during metastasis [45,82,83]. As for NOX5, a recent study combined gene expression and flux balance analysis of BRAF-mutant melanomas to reveal the upregulation of NOX5 expression that is associated with the resistance to BRAF inhibitor treatment [84]. Isoforms of the NOS family have also been found to be upregulated in melanoma and contribute towards elevated proliferation and progression [85,86]. Interestingly, ROSs are also produced under circumstances of intermittent hypoxia, and NRF2 has been reported to contribute to melanoma metastasis in a mouse model of sleep apnea [87]. Another cause for NRF2 activation is alterations in its regulation at the transcriptional and posttranslational levels, as implicated by its expression during progression.

### 2.4. Expression and Regulation of NRF2 in Melanomas

A histological examination of melanomas for NRF2 expression indicates that NRF2 expression decreases early in melanomagenesis between benign and dysplastic nevi and maintains that lower expression between primary and metastatic stages [88]. At the same time, however, nuclear NRF2 expression is an indicator of a poor prognosis correlating with reduced survival [88,89]. Interestingly, a recent study investigating changes in the melanoma proteome following NRF2 modulation revealed that the inhibition of NRF2 expression led to changes in the expression of genes related to melanoma progression including stem cell marker CD44 [90]. It was hypothesized that reduced NRF2 expression could contribute to phenotype switching exhibited in melanomas in which a proliferative state changes to an invasive state or vice versa [91]. This switching occurs in part via changes in the expression of MITF, the master transcriptional regulator of melanocyte differentiation, where upregulation leads to a proliferative phenotype and downregulation causing an invasive phenotype [92]. It could be that NRF2 expression is needed during periods of rapid growth and is downregulated when an invasive phenotype is established, but more research is needed to confirm this potential relationship.

Despite reduced levels of NRF2 expression during melanoma progression, there is continued expression of NRF2 transcriptional targets. This continued transcription could be due to the contribution of other transcription factors, such as NF-κB, or the altered regulation of NRF2 allowing transactivation activity even with a reduced amount of the protein. In a subset of melanomas, this is explained by *KEAP1* deactivating mutations that have been observed and cause elevated NRF2 expression following transformation to a primary tumor [93].

Other regulators include the homologous histone acyltransferases p300 and CBP that function as co-activators promoting the transactivation activity of NRF2. It was previously shown that p300/CBP acetylate NRF2 which enables NRF2 to bind to ARE sequences and promote expression of target genes [94]. The expression of the p300 gene, *EP300*, is often upregulated, as it is located in regions of chromosomal copy gain seen in melanomas [95]. Interestingly, a histological analysis of melanomas revealed that, throughout progression, p300 nuclear localization decreases and cytoplasmic localization increases [96]. Furthermore, the reduced nuclear expression of p300 correlated with a poorer prognosis in patients. This could be due to p300/CBP contributing to MITF transcriptional activity, and so these changes could be another result of phenotypic switching [97]. Evidence of this comes from a study into the pharmacologically inhibition of p300/CBP, which leads to reduced proliferation and MITF transcriptional activity [98]. It is likely that this occurs as melanomas progress to a more invasive phenotype as seen in the histological data. The knockdown of p300/CBP causes reduced growth, increased cell death, and an elevation in ROS, which further indicates their contribution towards a proliferative phenotype [99].

The RXRα nuclear receptor acts as a co-repressor of the binding of NRF2 to the Neh7 domain and has been shown to downregulate the expression of ARE-regulated genes [69]. A histological analysis of RXRα expression throughout melanoma progression indicates that its nuclear expression decreases between benign nevi and the primary tumor [100]. This lower expression continues during metastasis. We have shown, using a melanocyte-specific knockout mouse model, that the loss of RXRα and isoform RXRβ in melanocytes leads to reduced recruitment of IFN-γ secreting immune cells following UVR, due to the altered expression of intercellular signaling chemokines and cytokines [101]. It has also been reported that both NRF2 and ROS, in turn, negatively regulate RXRα expression, which might explain why it is reduced during melanoma progression. Possibly, this negative regulation could be connected to resistance to immunotherapy [102,103]. This is because IFN-γ positively modulates PD-L1 expression, and higher PD-L1 expression has been correlated with the improved efficacy of anti-PD-1 therapy [104,105]. NRF2 itself has also been reported to modulate PD-L1 expression directly, although this report conflicts with the previous studies by suggesting the combined inhibition of PD-1 and PD-L1 [106].

Overall, the regulation of NRF2 transactivation activity is highly complex, and further research is needed to understand how it contributes to the altered redox homeostasis seen in melanomas. It is likely that a combinatorial approach would be required if NRF2 itself were to be targeted, as it is likely that a monotherapy would be detrimental due to melanoma phenotype switching. It could be that any one of the ARE-regulated antioxidant gene products might prove a more effective therapeutic target due to the intricate web of interactions surrounding NRF2.

## 3. The Glutathione Antioxidant System

**Figure 3:**
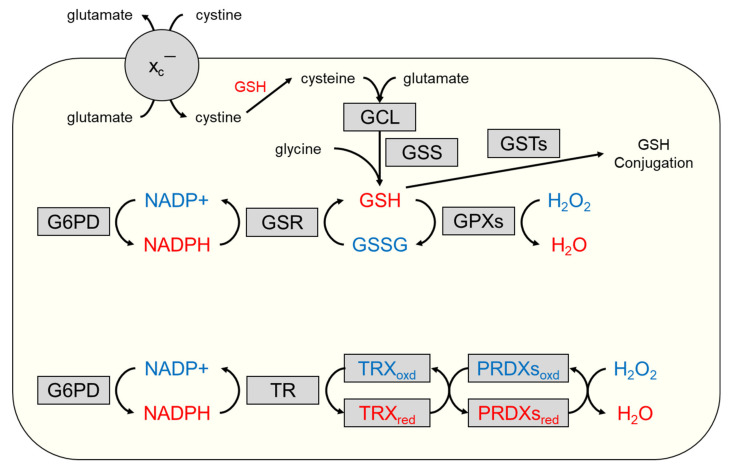
Schematic overview of the cysteine-dependent glutathione (top) and thioredoxin (bottom) antioxidant systems. The glutathione antioxidant system is comprised of the enzymes necessary for the synthesis, utilization, and recycling of the tripeptide glutathione. The amino acid cysteine is essential to both the glutathione and thioredoxin antioxidant systems and is taken into the cell in the form of the disulfide dimer cystine by the cystine/glutamate antiporter xCT (system x_c_^−^), the protein subunits of which are encoded by NRF2 transcriptional targets *SLC7A11* and *SLC3A2*. Cystine is rapidly reduced into cysteine in cytosol to be further utilized by the cell. The thioredoxin antioxidant system is comprised of the enzymes necessary for the utilization and recycling of the thioredoxin enzyme. Proteins in grey shapes are encoded by genes directly regulated by NRF2 through AREs. The blue text in the figure indicates the oxidized form of a molecule/protein, and the red text indicates the reduced form. ARE, antioxidant response element; G6PD, glucose-6-phosphate dehydrogenase; GCL, glutamate cysteine ligase; GPX, glutathione peroxidase; GSH, glutathione GSR, glutathione reductase; GSS, glutathione synthetase; GST, glutathione-S-transferase; PRDX, peroxiredoxin; TR, thioredoxin reductase; and TRX, thioredoxin.

The glutathione (GSH) antioxidant system consists of the enzymes necessary to synthesize, utilize, and recycle GSH (the predominant non-protein thiol) to regulate the level of ROS present in the cell (Figure 3). GSH is a tripeptide comprised of sulfur-containing cysteine, glutamate, and glycine in the form of γ-L-glutamyl-L-cysteinylglycine [107]. The enzymes involved in its synthesis are the rate-limiting glutamate cysteine ligase (GCL), comprised of catalytic (GCLC) and modifier (GCLM) subunits, and GSH synthetase (GSS) [107]. The GSH synthesis enzyme genes *GCLC*, *GCLM*, and *GSS* have AREs in their promoter regions and are positively regulated by NRF2 [108,109,110].

GSH is largely localized to the cytosol but is also present to a lesser extent in the mitochondria and endoplasmic reticulum [111,112]. Key to the function of GSH as an antioxidant is that it provides electrons for selenium-containing glutathione-dependent peroxidases (GPXs), which go on to reduce both hydrogen peroxide and lipid peroxides [107]. In this process, GSH is oxidized to its disulfide form (GSSG). The recycling of GSSG back into its reduced form is catalyzed by glutathione reductase (GSR) and consumes NADPH produced in part by glucose-6-phosphate dehydrogenase (G6PD) in the pentose phosphate pathway [107]. Of the genes encoding these enzymes, *GPX2*, *GSR*, and *G6PD* are known to have promoter regions containing AREs and are regulated by NRF2 [48,113,114].

Another key family of enzymes in this system are glutathione-S-transferases (GSTs), which are split into three overarching categories: microsomal, mitochondrial, and cytosolic. Cytosolic GSTs are further divided into seven classes in humans, with pi and mu being the most studied [115]. GSTs are phase II enzymes responsible for detoxifying, via GSH conjugation, electrophilic molecules that can include anticancer drugs, which often implicates GSTs in chemoresistance [115]. GSTs are often upregulated in melanomas, as with many cancers [116]. Both *GSTP1* and *GSTM1* are known to be regulated by NRF2 through ARE binding [117].

Glutathione synthesis genes have been found to be upregulated in many cancers including melanoma [54,118]. However, it has also been reported that the expressions of *GCLM* and *GCLC* do not correlate with NRF2 expression in melanoma [118]. This is interesting, as NRF2 expression has been reported to decrease with melanoma progression, as previously discussed. It has also been determined that melanomas with higher expressions of GCLC correlate with a better prognosis in patients; however, GCLC expression does not necessarily indicate changes in GCL activity, as GCLM expression plays a larger role in controlling the function of GCL [119,120]. Melanoma cells do appear to benefit from elevated cellular GSH content, as it has been shown to contribute towards melanoma cell proliferation and metastasis [51]. Indeed, it appears that GSH levels are depleted as melanomas progress to metastasis, and this helps to explain the potential detrimental effects of an exogenous antioxidant treatment [121]. Inhibition of glutathione synthesis via the GCLC inhibitor buthionine sulfoximine has not been shown to be effective against melanomas in vitro or in vivo; however, in combination with cysteine transport or thioredoxin inhibition, greater efficacy is achieved [52,122].

Expressions of *GPX2*, *GPX4*, *GSR*, and *G6PD* have been found to be elevated in melanomas and other cancers [54]. Interestingly, GPX3 seems to be downregulated in both melanoma cell lines and tissues due to promoter region hypermethylation [123]. In that same study, it was also determined that GPX3 overexpression inhibits melanoma proliferation, and silencing *GPX3* expression had the inverse effect on proliferation while promoting a more invasive phenotype. Furthermore, induced GPX3 expression in melanoma cells causes a metabolic shift towards oxidative phosphorylation and away from glycolysis, while also negatively regulating both HIF1-α and HIF2- α due to decreased stabilization in the relative absence of ROS [124]. GPX2 and GPX4 have been demonstrated to play a role in the resistance of melanoma to therapies [125,126]. There are some studies that have investigated blood GPX activity as a biomarker in melanoma patients, where increased activity is shown as compared to healthy volunteers or after surgical resection [127,128]. Pharmacological inhibition of GSR in melanoma cell lines has been shown to reduce proliferation, reduce migration, increase ROS, and decrease cellular GSH:GSSG ratio, suggesting the GSR function is co-opted by melanomas [129]. The authors of that study also found that inhibition of GSR induced oxidative stress that hindered melanoma cell metastasis, further indicating the co-option of endogenous antioxidant systems during disease progression. The role of G6PD in cancer redox biology, including for melanomas, has recently been reviewed [130]. Melanoma cell lines have been demonstrated to have elevated expressions and activities of G6PD compared with normal melanocytes, and the knockdown of G6PD in xenograft models show G6PD facilitates melanoma growth in vivo [83,131]. That same group also reported that G6PD works in tandem with NOX4 to activate STAT3 signaling to promote melanoma proliferation and avoid cell cycle arrest [83].

As the predominant cellular antioxidant, GSH and its associated enzyme effectors are co-opted during melanoma initiation, progression, and metastasis. A common theme seen by constituent enzymes in the GSH system is a contribution towards melanoma cell proliferation and its ability to metastasize. Furthermore, GPXs and GSTs are strongly associated with the development of drug resistance in melanomas and continue to hinder effective chemotherapeutic treatment. These factors highlight the altered redox homeostasis present in melanomas while providing potential targets for novel therapies.

## 4. The Thioredoxin Antioxidant System

The thioredoxin antioxidant system is comprised of thioredoxin (TRX or TXN), thioredoxin reductase (TR), and NADPH [132]. This system functions via the transfer of electrons from NADPH to TR, which can then go on to reduce TRX (Figure 3). Reduced TRX can then go on to modulate the redox status of a multitude of substrates that include peroxiredoxins, apoptosis signaling kinase 1, and DNA synthesis enzyme ribonucleotide reductase [133,134,135]. TRX has two isoforms, TR1 and TR2, which are expressed in the cytoplasm and mitochondria, respectively. Upon reducing a substrate, the two cysteine residues in the active site of TRX are reversibly oxidized to form a disulfide bond, and then, much like GSH and GSR, they can be recycled to a dithiol via TR to enable further antioxidant activity [132]. TR is a selenocysteine-containing, homodimeric flavoprotein. Two isoforms predominate in all tissues: cytosolic TR1 and mitochondrial TR2. A third isoform, TR3, is expressed in the testes [132]. In addition to reducing TRX, TR1 is also known to play a role in regulating the DNA-binding activity of the transcription factor, nuclear factor kappa B (NF-κB) [136,137]. As with the GSH antioxidant system, the enzymatic activity of G6PD in the pentose phosphate pathway is a source of NADPH that feeds the TRX antioxidant system [48]. Due to the elevated intracellular ROS present in cancer cells, the expression of the thioredoxin antioxidant system has been shown to be upregulated in many tumor types [138]. Both *TXN* and *TXNRD1*, encoding TRX and TR1, respectively, are known to contain AREs in their promoter regions and are directly regulated by NRF2 [139].

The expression of both *TXN* and *TXNRD1* is known to be elevated in melanomas and other cancers [54]. TRX has specifically been shown to have increasing expression throughout disease progression, and its overexpression contributes towards enhanced metastasis [140]. Furthermore, TRX secretion by melanomas has been demonstrated to contribute to cancer immunoevasion by the recruitment and differentiation of T cells into immunosuppressive regulatory T cells [140]. The negative regulator of TRX, thioredoxin interacting protein (TXNIP), has been shown to have a reduced expression from benign nevi to primary melanoma and a further reduced expression in metastatic melanoma [141]. Interestingly in that same report, TXNIP expression was elevated following treatment with the BRAF inhibitor, PLX4032, and then decreased again with acquired drug-resistance, indicating a potential role for TRX in that process. 

The expression of TR1 has been analyzed in melanoma patient-tissue microarrays revealing a continuous elevation in TR1 expression from benign nevi to primary melanoma and further into metastatic melanoma [53]. In that same study, it was also demonstrated that the knockdown of *TXNRD1* induces glycolysis dependency in melanoma cell lines, and that the combination of *TXNRD1* knockdown with a pharmacological blockade of glycolysis-hindered metastasis in a mouse xenograft model. TR1, acting through TRX, has also been directly implicated in cancer cell motility, where it has been shown to modify the Cys101 residue of L-plastin to promote migration. This would indicate a potential role for the TRX system in melanoma metastasis [142]. Indeed, in our study investigating the role of TR1 in normal melanocyte development, we observed a depigmentation phenotype in the bodily regions most distant from the developmental tissue of origin for the melanocyte lineage, the neural crest, which further supports a role for the TRX system in cellular migration [43]. The inhibition of TR1 via auranofin or the small molecule MJ25 has demonstrated some antimelanoma efficacy in vitro, where they exhibited cytotoxicity, and MJ25 weakly induced p53 expression and activity [143]. However, targeting TR1 for melanoma treatment alone is unlikely to lead to any therapeutic outcomes due to the ability of compensatory antioxidant mechanisms to be engaged via NRF2 activation [43,137]. As mentioned previously, the elevated nuclear localization of NRF2 has been an indicator of poor prognosis in melanomas, and so any treatment that might activate NRF2 could be a risk if the cancer persists after treatment. It is likely that TR1 inhibition would need be combined with approaches that involve the interruption of the GSH system or cysteine transport into the cell in addition to targeted or immunotherapies. 

Similar to the GSH system, the function of TRX and TR1 are integral to normal melanocyte physiology, and their functions are co-opted by melanoma. It appears that the TRX system contributes towards progression by aiding in both proliferation and immunoevasion. The system also contributes to metastasis through involvement in cell motility and appears to be involved in drug resistance. 

## 5. Cystine/Cysteine Cycling

Cysteine, a reduced form of the disulfide dimer cystine, is a crucial amino acid that is a constituent of many cellular antioxidant proteins due to its capability to undergo redox reactions [107,132]. Therefore, the ability of cells to import cystine is crucial for regulating oxidative stress. This is accomplished through the cystine/glutamate antiporter xCT (system x_c_^−^) that functions via the exchange of intracellular glutamate 1:1 for extracellular cystine [144]. Imported cystine is then rapidly reduced in the cytosol to cysteine, where it can be utilized by the cell or exported back into the extracellular space to provide a reducing environment [145]. Two genes encode the subunits of system x_c_^−^, *SLC7A11* (xCT) and *SLC3A2* (4F2) [146]. *SLC7A11* encodes the functional, specific subunit of system x_c_^−^, while *SLC3A2* encodes a subunit that comprises multiple amino acid transporters. As an important source of cysteine necessary for intracellular and extracellular antioxidant activities, *SLC7A11* is upregulated in many cancer types in order to alter normal redox homeostasis and mitigate the cytotoxic effects of elevated oxidative stress [147]. The promoter region for *SLC7A11* is also known to contain an ARE and to be directly regulated by NRF2 [148].

*SLC7A11* expression has been shown to be elevated in cancers such as melanoma, both before and after acquired drug resistance [54,84]. The cysteine cycle has recently been identified as a potential target for melanoma therapy, as inhibiting the function of xCT with (S)-4-carboxyphenylglycine has demonstrated melanoma cell cytotoxicity, especially in combination with inhibition of GSH synthesis using buthionine sulfoximine [52]. Furthermore, the xCT antiporter has been demonstrated to contribute towards both cell-line and mouse xenograft-melanoma proliferation, and the small molecule riluzole has been reported to be an inhibitor [149]. *SLC7A11* CRISPR/Cas9 knockout inhibits proliferation, migration, and invasion in vitro, and mouse xenograft experiments demonstrated that cysteine transport also facilitates proliferation and metastasis in vivo [150]. *SLC7A11* has also been shown to be upregulated in BRAF inhibitor-resistant melanoma cell lines, which themselves exhibited elevated oxidative stress as compared to drug naive cells [151]. It was also determined that vorinostat, a histone deacetylase inhibitor, treatment after MAPK inhibition suppressed *SLC7A11* expression in patients with metastatic melanoma. Together, these studies highlight the potential for targeting *SLC7A11* in combination with other therapies to counteract the altered redox homeostasis present in melanomas both before and after acquired drug resistance.

## 6. Peroxiredoxins and Sulfiredoxin

Peroxiredoxins (PRDXs) are a family of antioxidant enzymes key in regulating intracellular oxidative stress via the level of hydrogen peroxide in the cell [152]. There are six mammalian isoforms (PRDX1-6) that contain an active site with either one or two redox-active cysteine residues. PRDX isoforms also differ in subcellular localization with PRDX1 and PRDX2 in the cytosol and nucleus; PRDX3 in the mitochondria; PRDX4 in the ER (also exists extracellularly); PRDX5 in the mitochondria, peroxisomes, and cytosol; and PRDX 6 in the cytosol [153,154,155,156,157]. The TRX and GSH antioxidant systems are capable of recycling oxidized PRDX, which allows it to continue its redox activity [152]. PRDXs have been found to be upregulated in numerous cancer types; however, only *PRDX1* and *PRDX6* have confirmed AREs in their promoter regions [139,156,158,159].

Sulfiredoxin (SRXN) is another redox enzyme that assists PRDXs in their antioxidant function. Particularly, SRXN is responsible for reducing hyperoxidized PRDX that can occur if the TRX or GSH systems are not able to reduce oxidized PRDX in time [152]. This hyperoxidation only occurs in the PRDXs that contain two active site cysteines, and so they do not interact with PRDX5 and PRDX6 [160]. The *SRXN1* isoform is known to have a promoter region ARE and to be regulated by NRF2 [161].

PRDXs 1-6 and *SRXN1* have been reported to have elevated expression in melanomas and other cancers [54]. However, there are conflicting reports of the decreased expressions of PRDX1 and *PRDX2* in at least a subset of melanomas. For PRDX1, histological staining revealed a loss of expression during the progression between benign nevi and malignant melanomas [162]. The two above contradictory reports could be due to the difference in expression level noted globally at the level of the transcript [54] vs. alteration in protein expression [162] noted in specific cell types within benign and advanced stage melanomas. Interestingly, mitochondrial PRDX1 expression appears to be upregulated in BRAF inhibitor-resistant cell lines, indicating a potential role in drug resistance [163]. Downregulation of *PRDX2* expression is known to occur due to the methylation of its promoter region; however, the cause for PRDX1′s reduced expression is not as clear [164]. PRDX2 has been shown to inhibit melanoma proliferation and migration in cell lines and metastasis in a mouse xenograft model [165]. This activity occurred due to the increase E-cadherin expression normally lost during melanoma progression. 

In uveal melanoma, the presence of cancers overexpressing PRDX3 has been an indicator of poor prognosis, with high expression correlated to metastasis and reduced patient survival [60]. PRDX6 has been associated with promoting melanocyte proliferation with expression either induced with EGFR stimulation or reduced with EGFR inhibition using erlotinib. SRXN has been found to have induced expression in mouse skin following tumor-promoting 12-O-tetradecanoylphorbol-13-acetate treatment and to also have elevated expression in human melanoma tissue microarrays [166]. It has also been shown to be upregulated after either TR1 inhibition via auranofin or siRNA knockdown of the melanocortin 1 receptor of melanoma cell lines [167,168]. Overall, the roles played by PRDXs in melanoma are complex, likely due to their distribution, and further research into the role they play is needed if they are to be targeted for melanoma therapy. SRXN1, on the other hand, appears to have a clearer role in promoting melanoma initiation; however, its exact role in melanoma progression and metastasis still needs to be elucidated.

## 7. NAD(P)H: Quinone Oxidoreductase 1

NAD(P)H: Quinone Oxidoreductase 1 (NQO1) is a phase II antioxidant flavoenzyme that detoxifies via the reduction of reactive quinones to hydroquinones in a manner that avoids generation of ROS [169]. It is largely localized to the cytosol and exhibits low and consistent expression under normal conditions [170]. In the skin, the expression of NQO1 can be induced by UVR, particularly the UVA wavelength, to protect against oxidative stress [41,171]. Other important functions of NQO1 include the stabilization of p53 during conditions of elevated DNA damage, as well as regulating the initiation of 20 s proteasomal degradation [172,173]. In cancer, NQO1 is often highly expressed relative to normal tissue and there is ongoing research into therapies involving NQO1 substrate pro-drugs used to selectively target cancer cells [169,174]. *NQO1* is well known to be regulated by NRF2 and has a confirmed ARE sequence in its promoter region [175].

As with many other NRF2 antioxidant transcriptional targets, NQO1 has been shown to be upregulated in melanomas and other cancers [54]. A histology of melanoma tissue microarrays indicates that NQO1 is upregulated in melanoma but has no association with patients’ five-year survival [176]. It was hypothesized that NQO1 might play a role in initiation while not notably affecting progression and metastasis. In a clinicopathological study of melanomas, the same group analyzed the expression of six biomarkers, including NOQ1, which were elevated in early stages (I and II) and reduced at later stages (III and IV) [177]. In melanoma cell lines, NQO1 has been demonstrated to promote proliferation by promoting cell-cycle progression [57]. Induced NQO1 expression has also been shown to induce melanogenesis in cancer cell lines, with silenced expression having the opposite effect [178]. It is possible that NQO1 is directly or indirectly associated with MITF activity in melanomas and, therefore, might be involved with a proliferative phenotype over an invasive one. 

## 8. Heme Oxygenase 1

Heme oxygenase 1 (HO-1) is an antioxidant enzyme that catalyzes the first rate-limiting step in the degradation of heme into the antioxidant biliverdin, carbon monoxide, and pro-oxidant ferrous iron [179]. The ferrous iron is then taken up by ferratin when is often upregulated at the same time as HO-1 [180]. Biliverdin is reduced by biliverdin reductase into bilirubin, which also engages in antioxidant activity. HO-1 expression is induced by elevated cellular oxidative stress, as seen in its induction following UVA radiation of the skin [41,171]. In cancer, elevated HO-1 expression upregulates proliferation, resistance to anticancer therapy, and immune evasion [181,182,183,184]. HO-1 expression also often favors cancer progression to metastasis [185]. As a phase II enzyme, *HMOX1* (encoding HO-1) is regulated by NRF2 and contains an ARE in its promoter region [186].

The elevated expression of *HMOX1* has been seen in multiple cancers that also include melanoma [54]. Overexpression of HO-1 in melanoma cell lines causes increased proliferation and increased resistance to oxidative stress, and xenograft experiments with overexpressing cell lines cause reduced survival in mice [187]. Together, these data indicate HO-1′s role in melanoma progression. HO-1 also has been shown to contribute towards dysregulated proliferation in BRAF^V600E^ melanomas, through direct association with the BRAF protein [58]. Furthermore, it has been implicated in BRAF inhibitor resistance where HO-1 silencing or inhibition, using tin mesoporphyrin IX, increased sensitivity to vemurafenib, increased IL-15-activated natural killer cells, and upregulated natural killer-cell target expression enabling the improved activity of melanoma cells [188]. HO-1 has also been shown to mediate the differential stress response between normal tissue and melanomas seen in the efficacy of therapy combining short-term starving or fast-mimicking diet and doxorubicin [189]. This occurs at least in part due to enhancing anti-melanoma immune cell accumulation. From these studies, it is evident that HO-1 plays an important role in melanoma disease progression and provides a promising target for overcoming BRAF inhibitor resistance going forward.

## 9. Heat Shock Protein 70

Heat shock protein 70 (HSP70) is a member of the heat shock protein superfamily, which act as molecular chaperones that assist in the correct folding, transport, and degradation of cellular proteins [190]. Thirteen HSP70 family members haven been identified so far and are encoded collectively by 17 genes enabling diverse subcellular localization for their function [191]. HSP70 proteins have been considered to engage in antioxidant activity due to their ability to protect cells against oxidative stress, possibly through their ability to sequester damaged proteins and prevent aggregation [192,193,194]. The expression of HSP70 is upregulated in numerous cancer types and has been shown to limit apoptotic signaling through the PI3K-Akt pathway. HSP70 does so by binding and stabilizing Akt and inhibiting Bax activation [195,196]. An ARE has been shown to be located in the promoter region of *HSP70*, and so its expression can be regulated by NRF2 [197]. 

HSP70 has been shown to have elevated expression in melanoma tissue microarrays, when compared with benign nevi, and is also known to protect melanoma cells from oxidative stress, such as that following UV irradiation [198,199]. The inhibition of HSP70, via the 2-phenylethyne-sulfonamide derivative PET-16, resulted in melanomas that exhibited reduced migration and invasion through direct association of HSP70 with both FAK and BRAF^V600E^ [198]. A natural product source for potential HSP70 inhibitors is the C-prenylated chalcone, isocordoin 1. Derivatives of isocordoin 1 have been demonstrated to be cytotoxic, induce apoptosis, and decrease expression of HSP70 in melanoma cells lines [200].

There is also a potential role for using melanoma “cell surface expressed HSP70” to improve immunotherapy by triggering natural killer cell targeting to induce cancer cell death [201]. One route that has been studied for presenting melanoma expressed HSP70 to the immune system is through extracellular vesicles. Incubation of treatment-naive B16 melanoma cells with either exogenous soluble HSP70 or exogenous extracellular vesicles loaded with EVs led to the increased melanoma-cell surface expression of HSP70 that led to immune response-mediated melanoma-cell death [202]. In that same study, mouse xenograft models using B16 cells treated with HSP70-loaded EVs also exhibited reduced tumor volume and increased survival, suggesting that the induction of melanoma-cell surface expression of HSP70 could aid immunotherapy. 

## 10. Superoxide Dismutase (SOD)

The superoxide dismutase (SOD) family of enzymes regulate both extracellular and intracellular ROS-mediated oxidative stress [50]. They do so by converting superoxide into hydrogen peroxide, which can be further processed by other antioxidant enzymes. There are three human SOD genes: *SOD1* (copper/zinc SOD), *SOD2* (manganese SOD), and *SOD3* [50]. The three SOD enzymes have distinct localizations, with SOD1 expressed in the cytosol, SOD2 in the mitochondria, and SOD3 expressed extracellularly [203,204,205]. Overall, the SOD enzymes do not tend of be overexpressed in cancer and often exhibit lower expressions than normal tissue [205]. All three SOD enzymes have been shown to be regulated by NRF2; however, only *SOD1* and *SOD2* have a confirmed ARE in the promoter region [206,207,208].

Gene expression analysis of melanomas and other cancers indicated an upregulation of *SOD2* expression, while a histological examination of pigmented melanoma tissue samples revealed the upregulated expression of both SOD1 and SOD2 compared to healthy skin [54]. Interestingly, mimicking SOD2 with the mitochondrial superoxide scavenger (2-(2,2,6,6-tetramethylpiperidin-1-oxyl-4-ylamino)-2-oxoethyl) triphenylphosphonium chloride induced melanoma-cell line death and apoptosis and reduced tumor size in mouse xenografts [209]. However, SOD also appears to play a role in BRAF-inhibitor resistance in melanoma cell lines as BRAF inhibition of drug-naive melanoma cell lines induces elevated free radical formation. Supplementation of treated cells with SOD or inhibiting nitric oxide synthase counteracts the effectiveness of BRAF-inhibitor treatment and rescues the growth of *BRAF*-mutant melanoma cells [210]. After the development of BRAF inhibitor resistance, melanoma cell lines exhibit elevated levels of ROS and expression of SOD2 [211]. In that same study, the knockdown of SOD2 expression in resistant melanoma cells inhibited proliferation, while the administration of the exogenous antioxidant, N-acetyl cysteine, re-sensitized resistant cells to BRAF-inhibitor treatment. These studies indicate that SODs, especially SOD2, are promising targets for overcoming drug resistance by disrupting antioxidant protection of the mitochondria.

## 11. Clinical Applications of Exogenous Antioxidants

With the knowledge that melanomas are associated with elevated oxidative stress, clinical investigators have aimed to find therapeutic candidates that can pursue this feature as an avenue for a novel method of intervention against and/or treatment of melanoma. Naturally, this has led to investigation of dietary and topical antioxidants, such as vitamin A derivatives, vitamin C, vitamin E, nicotinamide, and selenium, for the prevention and treatment of melanoma. The results of these studies help highlight the complexity of the altered redox homeostasis present in melanomas that complicates their treatment with exogenous antioxidants.

### 11.1. Vitamin A

Clinical studies suggest that retinoid or carotenoid use in the context of melanoma prevention appears to have either a slightly protective effect or no effect on risk. In the Vitamins and Lifestyle (VITAL) study, a large prospective cohort of 69,635 participants taking retinol supplements showed a decreased risk of melanoma (HR = 0.60, 95% CI 0.41–0.90); however, they found that a dietary or total intake of vitamin A or carotenoids was not associated with melanoma risk [212]. Prospective data from the Nurses’ Health Study showed that increased retinol intake from foods and supplements appeared protective within a subgroup of women who were otherwise at low risk for melanoma based on several non-dietary factors, with RR = 0.39 and 95% CI of 0.22–0.71; in other groups, there was no significant effect [213]. On the other hand, the Women’s Health Study, a randomized, double-blind, placebo-controlled trial, reported that beta carotene use had no impact on melanoma risk, with a relative risk of 0.90 (95% CI 0.49–1.68) [214]. A Meta-Analysis by Zhang et al., which included a total of eight case-control studies and two prospective studies, suggests that the intake of retinol, rather than total vitamin A or beta-carotene, is significantly associated with a reduced risk of melanoma, with a summary odds ratio for the highest compared to lowest intake of retinol, total vitamin A, and beta-carotene found to be 0.80 (95% CI = 0.69–0.92), 0.86 (95% CI = 0.59–1.25), and 0.87 (95% CI = 0.62–1.20), respectively [215]. Additionally, several clinical trials have reported the histologic and clinical improvement of dysplastic nevi with the use of topical tretinoin (all trans-retinoic acid) [216,217].

### 11.2. Vitamin C

In pre-clinical studies, vitamin C, a well-known antioxidant, has been shown to induce the apoptosis of malignant melanoma cells at higher concentrations but may promote tumor growth at lower concentrations [218]. However, there is very little clinical evidence regarding vitamin C and its potential effects on the prevention and/or treatment of melanomas. Prospective data from the Nurses’ Health Study showed that, contrary to preconceived expectations, a higher risk of melanoma was observed in those with greater dietary intakes of vitamin C with RR = 1.43 and 95% CI 1.01–2.00 and a significant positive dose-response with the frequency of orange juice consumption (*p* = 0.008) [213]. However, it is possible that another component in vitamin-C containing foods such as orange juice may be contributing to an increase in risk. In contrast, Murray et al. found that the application of a topical formula containing 1% alpha-tocopherol, 15% L-ascorbic acid, and 0.5% ferulic acid at the dose of 2 g/cm daily for four days resulted in decreased UV-induced p53 induction, cyclobutane pyrimidine dimers, and cytokine expression in vivo [219].

### 11.3. Vitamin E

Vitamin E exhibits strong antioxidant effects with its ability to quench free radicals and inhibit lipid peroxidation; thus, it is thought to have potential anti-cancer applications [220]. However, clinical studies regarding vitamin E’s effects on melanoma have had varied results. Several case control studies examining the effects of vitamin E on melanoma incidence evaluated in a 2020 review in *Cancer* have shown inconsistent results [217]. Prospective data from the Nurses’ Health Study showed neither total nor dietary vitamin E was associated with melanoma risk (multivariate RRs 1.11 (95% CI = 0.66–1.85) and 0.88 (95% CI = 0.59–1.32), respectively) [213]. Whereas the SU.VI.MAX trial, a randomized, placebo-controlled trial investigating the health effects of antioxidant vitamins and minerals, found that women consuming antioxidant supplements, including dietary supplementation with vitamin E, were at an increased risk for developing melanoma [221]. Studies involving the use of topical vitamin E appear to have more favorable effects. A 2002 in vivo study showed that topical vitamin E and N-ac15ysteinestein treatment 24 h prior to UV exposure significantly decreased the expression of matrix metalloprotease MMP-12 in UV-irradiated skin, and thus, may provide a protective effect [222]. As described above in the section on vitamin C, a topical formula containing 1% alpha-tocopherol, 15% L-ascorbic acid, and 0.5% ferulic acid may confer anti-cancer effects [219].

### 11.4. Nicotinamide (Niacinamide)

Nicotinamide is a member of the vitamin B3 group and has been shown to enhance the repair of both oxidative and UV-induced DNA damage in human melanocytes in pre-clinical studies [223]. However, there is little clinical evidence regarding nicotinamide and melanoma. The ONTRAC study (Oral Nicotinamide To Reduce Actinic Cancer) was a multicenter, phase III, double-blinded placebo-controlled trial in which 386 participants were recruited [224]. The ONTRAC study revealed a decreased rate in the number of new non-melanoma skin cancers in the nicotinamide group vs. placebo group; and, while the study did not aim to assess the effect of nicotinamide on the development of melanomas, 10 melanomas arose during the intervention period. The melanomas were evenly distributed between the treatment and control groups and showed no significant differences in thickness and invasiveness between the two groups [224]. No conclusions can be drawn regarding the association between nicotinamide intake and melanoma development, as recruitment of patients in the ONTRAC study was not based on their risk for melanoma, and there were very few cases of melanomas among participants [223].

### 11.5. Selenium

Selenium is thought to play a role in several cellular pathways with downstream anti-tumor effects; these effects include reducing DNA damage and oxidative stress, decreasing inflammation, enhancing the immune response, altering DNA methylation, regulating the cell cycle, inducing cell death of malignant cells, and inhibiting angiogenesis [225,226,227,228,229,230,231,232,233,234]. Selenium is also critical to the function of thioredoxin reductase and glutathione peroxidases, making it an important micronutrient for the body’s endogenous antioxidant systems [235]. It would then be reasonable to conclude that it could be beneficial for the prevention and treatment of melanomas; however, the clinical data available regarding selenium and its effects on melanomas and other skin cancers vary.

One prospective study performed in Poland reported the 10-year-survival of 375 melanoma patients depending on their serum selenium levels [225]. Patients were assigned to one of four groups depending on a one-time serum selenium level collected prior to treatment (other than initial resection) and were followed up from the date of melanoma diagnosis until death or 2020. They found that the subgroup with low serum selenium (<76.44 μg/L) was associated with an increased mortality rate in the 10 years following diagnosis compared to patients with higher selenium levels, HR = 8.42; *p* = 0.005 and HR = 5.83 and *p* = 0.02, for uni- and multivariable models, respectively [225]. Additionally, a separate cohort study of 200 patients with non-metastatic melanomas showed that patients with stage I and II melanomas with recurrence within two years had significantly lower serum selenium levels [236]. In contrast, an Italian cohort with high environmental exposure to selenium was found to have a significant 3.9-fold increase in melanoma incidence as compared to an unexposed cohort [237]. A 2018 Cochrane review also suggests that selenium supplementation may increase melanoma risk [238]. They found three randomized controlled trials that investigated the risk of melanoma following selenium supplementation, but only two were deemed to have a low risk of bias [238,239,240]. When only including the low-risk-of-bias studies, they found a relative risk estimate of 1.35 with a 95% confidence interval between 0.41–4.52 [238]. However, it should be noted that there were only eight cases in the selenium groups and four cases in the placebo groups. Whereas a 2016 meta-analysis reports that high selenium exposure neither increased nor decreased the risk of skin cancer based on six estimates from four studies (pooled OR = 1.09, 95% CI: 0.98–1.21) [241]. However, they suggested that high selenium exposure may have different effects depending on the type of cancer, stating that it decreased the risk of breast, lung, esophageal, gastric, and prostate cancer. It needs to be remembered that the meta-analysis of multiple studies performed under different conditions with multiple variables is distinct from specific clinical study/studies performed on a bigger cohort with well-controlled variables and following the adjustment of the confounding factors. 

Overall, exogenous antioxidants do not have a clear therapeutic role in the prevention or treatment of melanomas, as many of the available studies contradict one another, some even suggest that certain antioxidants could increase melanoma risk. These varying data further underscore the need to better understand the complex interplay between melanoma and both intracellular and microenvironmental oxidative stress. 

## 12. Conclusions and Future Directions

The elevated oxidative stress seen in melanomas has been of consistent interest as a potential source for therapeutic interventions. However, it is becoming increasingly clear that the notion that therapies need only to reverse increased oxidative stress is an oversimplification of the complex altered redox homeostasis present in melanomas. Indeed, there are numerous studies that demonstrate that exogenous antioxidant treatment of melanomas might be detrimental [62]. This is the case, due to the fact that oxidative stress hampers the ability of melanoma cells to metastasize, and those cells that have already metastasized possibly upregulate antioxidant systems to favor their survival and protect against the cytotoxic effects of oxidative stress [121].

In this review we have highlighted how protective endogenous antioxidant systems are co-opted during melanoma initiation and progression. The net effect of this co-option is an increased antioxidant capacity within melanoma cells which allows for their unrestrained proliferation, metastasis, and drug resistance [84]. Fortunately, this reliance on endogenous antioxidant systems for progression and metastasis reveals potential targets for novel therapies. This is seen in the glutathione antioxidant system, where there is upregulation of GSH synthesis genes, increased recycling of GSH mediated by GSR, and increased expression of endogenous antioxidants associated with drug resistance, such as GPX2, GPX4, and GSTs [54]. Furthermore, NRF2 contributes to the elevated expression of G6PD, which is an important source for NADPH necessary for the activity of both glutathione and thioredoxin antioxidant systems [54]. With the thioredoxin antioxidant system, there is an upregulation of both *TXN* and *TXNRD1* expression that contributes to PRDX function and the clearance of cellular free radicals [54]. Both of these systems are dependent on the amino acid cysteine, which is imported through the cystine/glutamate antiporter xCT (system x_c_^−^) comprised in part by the NRF2 transcriptional target, *SLC7A11*, which also has upregulated expression in melanomas [54]. 

Three potential antimelanoma targets within these antioxidant systems could include (but are not limited to) glutathione synthesis, thioredoxin reductase enzymatic activity, and the cystine/cysteine cycle. A combination of these redox targets would then be included with conventional melanoma therapies. For example, it has previously been shown that the combined inhibition of GSH synthesis and cystine/cysteine cycling was more effective in killing melanoma cells than either treatment alone [52]. This blockade of co-opted antioxidant function could then be combined with MAPK inhibition or immunotherapy. The knockdown of thioredoxin reductase 1 alone in melanoma alone does not prevent metastasis but does induce a dependency on glycolysis, and a complete knockout of *TXNRD1* in melanocytes increases the nuclear localization of NRF2 and the synthesis of GSH post-UVB irradiation [43,53]. Interestingly, acquired resistance to BRAF inhibition induces a shift to oxidative phosphorylation [242]. So, it would be interesting to determine the effect that combinatorial inhibition of MAPK signaling and thioredoxin reductase 1 would have on melanoma cells.

Another avenue of treatment through intracellular redox manipulation is through the targeting of mitochondria. We recently performed a high-throughput screen to identify compounds effective against drug-resistant melanoma and found that compounds that target the electron transport chain were the most potent and others have studied different compounds that act similarly [81,243]. Inhibition of the electron transport chain leads to the accumulation of oxidative stress, making it a pro-oxidant treatment [244]. Another promising avenue is compounds that are capable of disrupting mitochondrial membrane potential as exhibited by natural product polyphenols acting as proton shuttles that interfere with the electrochemical gradient across the inner mitochondrial membrane [245,246]. Additionally, polyphenols can also inhibit the electron transport chain and, thereby, further increase the oxidative stress present in cancer cells.

It is also important to examine NRF2 and its transcriptional targets in the context of the immune response. Melanoma cell NRF2 has an indirect connection with an immune response through its negative regulation of the transcription factor RXRα, where we have previously shown that knockout of RXRα and RXRβ in melanocytes alters the expression of the cytokines/chemokines that recruit IFN-γ secreting immune cells [101]. However, the targeting of NRF2 directly in an attempt to improve an antimelanoma immune response is not likely to be effective, as it has been previously shown that NRF2-null mice have an increased susceptibility to xenografts of B16-F10 melanoma cells, possibly due in part to an impaired immune system [247]. These data indicate that any intervention that might also compromise the NRF2 function in nonmelanoma cancer-adjacent cells could be detrimental, and, therefore, endogenous antioxidants downstream of NRF2/KEAP1/ARE signaling could be better targets of immunotherapy. These targets could include thioredoxin secretion, heme oxygenase 1, and cell-surface expression of heat shock protein 70, previously shown to play a role in the immune response to melanomas, as discussed above.

It should be noted that there has been some efficacy seen in clinical studies investigating the role of exogenous antioxidants in the prevention of melanoma, but its utility in treatment requires further detailed studies on intervention at different stages of melanoma progression and metastasis to better understand and subsequently avoid any unintended detrimental effects.
